# Antioxidant and Antiapoptotic Properties of n-Butanol Fraction the *Acanthopanax senticosus* Extracts in H_2_O_2_-RAW264.7 Cells and CCl_4_-Induced Liver Injury in Mice

**DOI:** 10.1155/2023/9190198

**Published:** 2023-02-23

**Authors:** Jianqing Su, Xiang Fu, Ruixue Liu, Xinyu Zhang, Jiaojiao Xue, Ying Li, Rui Zhang, Xiaoli Li, Xiaoya Wang, Xueyan Wang, Yi Ding, Xiuling Chu

**Affiliations:** College of Agronomy and Agricultural Engineering, Liaocheng University, Liaocheng 252000, China

## Abstract

The *Acanthopanax senticosus* has been shown to have a wide range of pharmacological activities, which are associated with health benefits, such as antioxidant, anti-inflammatory, and antiapoptotic properties. A previous study has shown that the n-butanol fraction of *A*. *senticosus* extract had the strongest antioxidant effect in vitro. This study aimed to investigate the effects that the n-butanol fraction of *A*. *senticosus* extract could relieve oxidative stress damage through antioxidant and antiapoptotic in the H_2_O_2_-stimulated RAW264.7 macrophages and the CCl_4_-induced liver injury. The result showed that the n-butanol fraction extract could relieve damage by increasing the intracellular antioxidant enzymes (SOD) level, decreasing intracellular ROS and MDA levels, and regulating antioxidant and antiapoptotic-related gene expression levels. The morphological observation of HE, TUNE, and immunohistochemistry staining of liver tissue verified that the n-butanol fraction extract is though anti-oxidative and antiapoptotic to alleviate cellular oxidative damage. The RT-PCR assay showed that the Keap1-Nrf2-ARE and the Bax/Bcl-2 signaling pathway were related to the molecular mechanism of action. The experimental results show that *Acanthopanax senticosus* extract has a good effect in treating liver injury and enhancing the antioxidant capacity of the body.

## 1. Introduction

Oxidative stress can produce a large number of reactive oxygen species (ROS) in the cell, and their prevalence occurs due to unbalanced oxygen homeostasis, leading to the production of intracellular oxidative stress [1], which can cause different degrees of oxidative damage and even apoptosis in the body and accelerate the process of cell disease [2, 3]. Studies have proved that the antioxidant capacity is inversely proportional to the ROS in vivo and that the strength of the antioxidant enzyme activity is closely related to the degree of oxidative stress. The decreasing antioxidant activity may directly lead to apoptotic [4].

Macrophages are important immunity cells for the host defense system to eliminate microbial pathogens. Studies have verified that macrophages, which can produce ROS as infected by some bacteria, are a suitable model for antioxidant research [5]. It was found that excess ROS are readily converted to H_2_O_2_ in cells and diffuse through cell membranes, undergoing a heme-catalyzed Fenton reaction to produce highly reactive and toxic hydroxyl radicals, which eventually trigger macrophage death. In contrast, the mouse macrophage cell line RAW264.7 is the most commonly used mouse macrophage cell line in medical research, so this study used RAW264.7 cells to establish an oxidative stress model. The liver is the main metabolic organ that converts glucose, fatty acids, and amino acids from the blood into the essential metabolic fuel for extrahepatic tissues during exercise and starvation [6], and the liver also is a detoxifying organ; thus, the liver is prone to oxidative stress. CCl_4_, a compound, can cause chemical liver damage, including inflammation, which is commonly used to induce acute and chronic liver injury in mice models [7].

*Acanthopanax senticosus* (Rupr. & Maxim.) Harms (AS), as traditional Chinese medicine, belongs to the genus *Acanthopanax* (Araliaceae), widely used in China, Korea, Japan, and Russia [8]. According to traditional Chinese medicine theory, AS can stimulate qi, strengthen the spleen, and nourish the kidneys [9]. The medicine chemistry studies showed that AS contains many pharmacological chemical compounds, including polysaccharides, saponins, polyphenols, and flavonoids [10]. Modern pharmacological experiments have demonstrated that *A*. *senticosus* possessed antistress, antioxidant, antiapoptotic, and hepatoprotective activities[11]. Previous works showed that the n-butanol fraction of *A*. *senticosus* extracts (NASE) has higher antioxidant activity in vitro [12]. As reported in the literature, n-butanol fractions of AS significantly prolong the swimming time of mice to exhaustion [13]. As a follow-up, this work aimed to explore the protective mechanism of NASE in the H_2_O_2_-induced RAW264.7 injury and the CCl_4_-induced liver injury in mice. This study provided theoretical support for NASE use in clinical settings.

## 2. Materials and Methods

### 2.1. Plant Materials, Chemicals, and Reagents

*A*. *senticosus* decoction pieces were purchased from Liaocheng Limin Pharmacy (batch no. 20200516). The CCK-8 assay kit, ROS testing kit, modified Giemsa staining solution, RIPA lysate kit, and BCA protein concentration detection kit were purchased from Beyotime Biotechnology. CCl_4_ was purchased from Jinan Chemical Co., Ltd.; ALT, AST, ALP, LDH, superoxide dismutase (SOD), and malondialdehyde (MDA) assay kits were purchased from Nanjing Jiancheng Biological Engineering Research Institute Co, Ltd.; Nuclear factor erythroid 2-related factor 2 (Nrf-2), Heme oxygenase-1 (Ho-1), Kelch-like ECH-associated protein 1 (Keap-1), NAD (P) H: quinone oxidoreductase-1 (NQO-1), Bcl-2, Bax, GAPDH primers were synthesized by Shanghai Sangon Biotechnology Co., Ltd. RAW264.7 cells were purchased from the Stem Cell Bank of the Chinese Academy of Sciences (Beijing, China).

### 2.2. Preparation of Extract of NASE

The NASE was prepared according to document 12. Briefly, the AS was extracted by a complex enzyme-assisted ultrasound method. The extraction conditions are enzyme ratio (cellulase: pectinase (3 : 2)), the amount of complex enzyme added is 6,960 U·g^−1^, hydrolysis time is 59.80 min, with a temperature of 53.70°C, and a pH value of 6.05. The active components in the extract were enriched and purified with different polar solvents. In the n-butanol fraction, the contents of total flavonoids and polyphenols were 61.0 ± 0.344 mg·g^−1^ and 24.93 ± 0.234 mg·g^−1^, being used for the following experiments.

### 2.3. Cell Culture, Cytotoxicity, and Cytoprotective Experiments

#### 2.3.1. Cell Culture

RAW264.7 cells were grown in Roswell Park Memorial Institute (RPMI) 1640 media supplemented with 10% heat-inactivated fetal bovine serum and 100 U/mL penicillin-streptomycin and maintained at 37°C in a 5% CO_2_ humidified incubator, and the media was refreshed once the other day and the cell was passaged once from two days to three days. When RAW264.7 cells were in a logarithmic growth phase reaching approx. 80% confluence and the cell was digested with pancreatic enzyme and washed twice with PBS (0.05 M, pH 7.2), the cells were prepared with a single-cell suspension of 1.8 × 10^5^ cell/mL and seeded in 96-well tissue culture plates at 200 *μ*L per well.

#### 2.3.2. Cytotoxicity Assay of NASE

The cells were divided into a normal group and different treatment groups. The treatment groups were treated with different concentrations of NASE (25, 50, 100, 150, 200, and 250 *μ*g·mL^−1^, which was diluted by 1640 medium). When the cells reached 80% confluence, the supernatant was removed and washed with PBS once, added 100 *μ*L of serum-free medium and 10 *μ*L of CCK-8 solution per well, continuing to maintain in the incubator for 2 h, the absorbance was determined at OD 450 nm using an ELISA reader and cell viability was calculated [14].

#### 2.3.3. Hydrogen Peroxide (H_2_O_2_) Toxicity Test

The H_2_O_2_ toxicity assay on RAW264.7 cells was also evaluated by the CCK-8 assay. When the cells were reached the logarithmic growth phase, and the cell density was adjusted to 5 × 10^4^ cells·mL^−1^, seeded the cells in 96-well plates, continuing to culture for 24 h. Then, the supernatant was discarded, by additional media with the final concentration of 100, 200, 300, 400, 500, 600, 700, 800, and 900 *μ*mol·L^−1^ hydrogen peroxide in a 96-well plate, and allowed to interact for 2 hours. The absorbance was determined at OD 450 nm according to the abovementioned method, and the cell survival rate was calculated.

#### 2.3.4. Cytoprotective Experiments

The cells were divided into a normal group, an H_2_O_2_ group, and three experimental groups, including the NASE low-dose group (25 *μ*g·mL^−1^), NASE medium-dose group (50 *μ*g·mL^−1^), and the NASE high-dose group (100 *μ*g·mL^−1^). After being added NASE for 24 h, the cell was added medium with H_2_O_2_ of a final concentration of 300 *μ*mol·L^−1^, continued to interact for 2 h, determined the absorbance at OD 450 nm according to the abovementioned method, and calculated the cell survival rate [15].

### 2.4. Detection of Intracellular ROS

RAW264.7 intracellular ROS were monitored by a ROS assay kit with a fluorescent probe (DCFH-DA). RAW264.7 cells were inoculated in a 6-well plate and treated as mentioned above. The cells were washed three times using PBS (0.05 M, pH 7.2). Then, the cells were stained with 1 : 1000 diluted DCFH-DA and incubated for 30 min at 37°C according to the manufacturer's instructions. DCF fluorescence was detected by an inverted fluorescence microscope with an excitation wavelength of 485 nm and an emission wavelength of 530 nm in the laboratory. The fluorescence intensity of each group was analyzed using the Image *J* software.

### 2.5. Giemsa Staining

RAW264.7 cells were inoculated in a 6-well plate and treated as mentioned above, and the Giemsa staining method was carried out according to the manufacturer's instructions. The apoptotic cells and apoptotic vesicles were dark purple-blue and were easily recognized under a light microscope. Three fields of view were selected for each picture, and the number of positive cells was calculated in each field among 100 cells, and the percentage of the number of positive cells was calculated as the positive index of apoptotic cells.

### 2.6. Flow Cytometry Assay

RAW264.7 cells were inoculated in a 6-well tissue culture plate and treated as mentioned above, apoptosis was detected according to the Annexin V-FITC/PI kit. Adding 10 *μ*L of the Annexin V to each group of cells, incubated for 10 min, adding another 5 *μ*L of PI, and after 5 min of incubation, flow cytometry was used to detect the apoptosis rate of each group.

### 2.7. Animal Study

Kunming mice (6 weeks old, male) were obtained from Jinan Pengyue Experimental Animal Breeding Co., Ltd. (Shandong, China). All mice were randomized and transferred to mouse cages in an air-conditional room (temperature 24–25°C, humidity 60%–65%) under a 12 h light/dark cycle. Each group contains eight mice. All mice were allowed water and standard chow ad libitum. The study is approved under the regulations of the Committee on the Ethics of Animal Experiments at Liaocheng University (permit number: 20200126).

The mice were randomized into five groups: (normal group, model group, NASE low-dose group (20 mg·mL^−1^), NASE medium-dose group (40 mg·mL^−1^), and the NASE high-dose group (80 mg·mL^−1^).

The model group and three NASE-treated groups were injected intraperitoneally into a soybean oil solution containing 20% CCl_4_ at a dose of 1 mL·kg^−1^ three times a week for 2 weeks. The normal group was injected intraperitoneally with soybean oil three times a week for two weeks. For the NASE-treated groups, NASE was given to mice once a day by Gavage with different dosages. For the normal and model groups, mice were given double distilled water to minimize the effects of the gavages' procedure [16]. The mice were weighed daily. Two weeks after drug administration, the mice were randomly selected for dissection.

On day 14, the mice were killed by cervical dislocation. The liver of the mouse was weighted and pathological changes were observed. Apart of the liver tissue was fixed in a 4% paraformaldehyde solution, and the remaining liver tissue was frozen in a −80°C refrigerator. The liver index (liver weight/body weight) was calculated.

### 2.8. HE Staining, Immunofluorescence Staining, and TUNEL Assay

The liver paraffin tissue sections were routinely made. Histological changes were examined by hematoxylin and eosin [*H* & *E*] staining. The protein expression of Bcl-2 and Bax in liver tissue was detected, respectively, with an immunohistochemical assay. The immunohistochemical streptavidin biotin complex (SABC) method was used under the instructions of the SABC kit. The TUNEL staining was operated according to the colorimetric TUNEL apoptosis Assay. The images were observed by light microscopy and an upright Olympus fluorescence microscope. The staining intensity of immunohistochemistry was evaluated using the Image *J* software.

### 2.9. The Content of ALT, AST, ALP, and LDH in Serum

After the animal experiment, the mice were sacrificed, whole blood was obtained and placed at room temperature for 6 h and the blood was centrifuged at 5000 r/min for 5 min. The serum was separated. The contents of ALT, AST, ALP, and LDH in the serum were determined using the corresponding kit.

### 2.10. The Content of SOD and MDA

The RAW264.7 cells and liver tissue were ground with PBS (0.05 M, pH 7.2), and the supernatant was isolated by centrifuging at 4000 × *g* for 10 min at 4°C, then the concentration of protein was determined by a BCA protein detection kit. The MDA and SOD contents were determined using a kit based on the manufacturer's instructions.

### 2.11. Quantitative Real-Time Polymerase Chain Reaction

Total RNA from RAW264.7 cells and liver tissues was extracted with TRIzol reagents (Invitrogen) according to the manufacturer's instructions. The RNA concentration was determined by Nanodrop software. Reverse transcription was conducted using the TaKaRa kit. The real-time PCR was carried out with a BeyoFast TMSYBR Green qPCR Mix kit, and the reaction parameters were as follows: 95°C 2 min, 95°C 15 s, and 56–60°C 30 s for a total of 30 cycles. RNA samples were conducted in triplicate and data were normalized to the GAPDH level for mRNA. The gene expression levels for mRNA were quantified by the delta-delta comparative cycle threshold (2^−ΔΔCt^) method. The primer sequences are shown in Table 1.

### 2.12. Statistical Analysis

The SPSS 22 software was used to analyze the data. A one-way analysis of variance (ANOVA) test was applied to compare mean values, and *p* < 0.05 was considered statistically significant. GraphPad Prism 8 was used to plot. All data were expressed as mean ± standard deviation.

## 3. Results

### 3.1. Cytotoxicity and Cytoprotective Experiments

**Figure 1:**
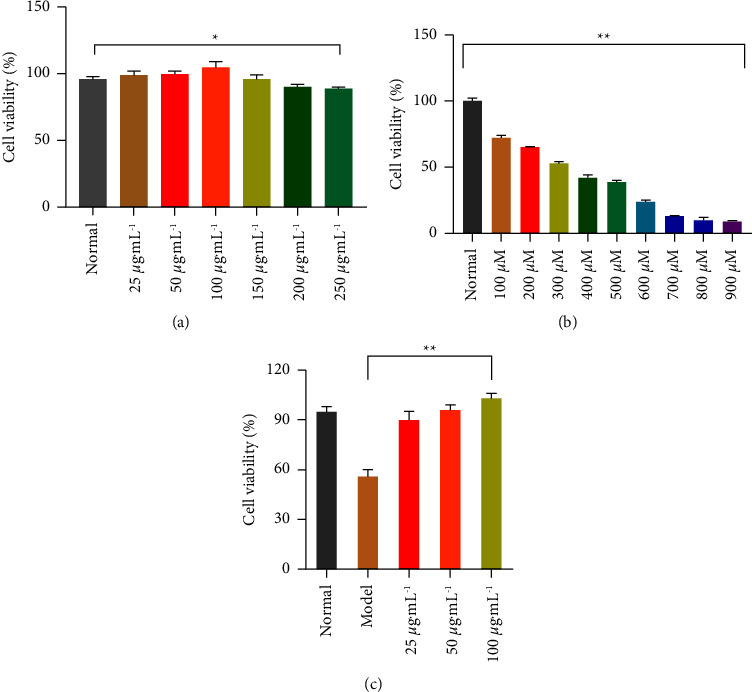
Cytotoxicity and cytoprotection assay. (a) The safe concentration of NASE on RAW264.7 cells. (b) The cytotoxicity effect of H_2_O_2_ on RAW 264.7 cells. (c) The protective effect of NASE on H_2_O_2_-induced RAW 264.7 cells. Note: Values are mean ± standard deviation. ^*∗*^Significant difference at the 5% level. ^*∗∗*^Extremely significant difference at the 1% level.

The RAW264.7 cells were treated with NASE at various concentrations in a 96-well plate. After interacting for 24 h, the ratio of cell survival was determined by the CCK-8 assay.  Figure 1(a) illustrates that NASE could promote cell proliferation within 25 to 100 *μ*g·mL^−1^ in a dosage-dependent manner. After reaching 150 *μ*g·mL^−1^, the survival rate of RAW264.7 cells decreased concerning the normal group (*p* < 0.05). It can be shown that the safe concentration of NASE is in the range from 25 *μ*g·mL^−1^ to 100 *μ*g·mL^−1^. To avoid the toxic effects of NASE on cells, the concentrations of NASE were selected as 25, 50, and 100 *μ*g·mL^−1^ in follow-up experiments.

The cytotoxicity of H_2_O_2_ on RAW264.7 cells was also evaluated by the CCK-8 assay kit. As shown in Figure 1(b), when its concentration reached 900 *μ*M, the survival rate of RAW264.7 cells was only 10.26 ± 1.23%, indicating that H_2_O_2_ stimulated RAW264.7 cells leading to oxidative damage and death. The IC_50_ of H_2_O_2_ on RAW264.7 cells was 290.7 *μ*M. To improve the operability of the experiment, 300 *μ*M was used as the subsequent treatment concentration.

The cytoprotection effect of NASE on H_2_O_2_-induced RAW264.7 cells was evaluated by the CCK-8 assay. According to Figure 1(c), the cell survival rate was significantly higher in the NASE group (25, 50, and 100 g·mL^−1^) than in the model group (*p* < 0.01). It appears that NASE reduces the oxidative stress damage caused by H_2_O_2_ and decreases cell mortality as a result. Thus, NASE could protect RAW264.7 cells against H_2_O_2_-induced oxidative stress damage.

### 3.2. Giemsa Staining

**Figure 2:**
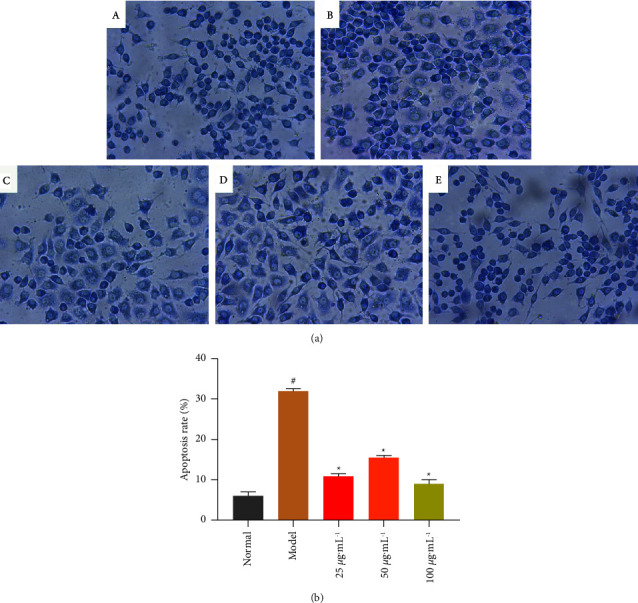
(a) Giemsa staining (40×). (b) Graph of the percentage of necrotic hepatocytes. (A) Normal group. (B) H_2_O_2_ group. (C) NASE low group dose (25 *μ*g·mL^−1^). (D) NASE medium dose group (50 *μ*g·mL^−1^). (E) NASE high dose group (100 *μ*g·mL^−1^). Note: Values are mean ± standard deviation. ^*∗*^Significant difference at the 5% level compared with the model groups. ^#^Significant difference at the 5% level compared with the normal groups.

The picture of Giemsa staining RAW264.7 cells was observed under a light microscope. As shown in Figure 2(a), the cell morphology of the normal group was bright and long spindle-shaped, with and uniform size of nuclei (Figure 2(a)). In the model group, the cells were shrunken and round after hydrogen peroxide injury, the nuclei were shrunken and the cell shape was not uniform (Figure 2(b)). In the NASE treatment groups, the cell morphology was significantly improved (Figures 2(c)–2(e)). Apoptotic cells can also be see in the picture, and the number of apoptotic cells decreased through NASE treatment in the NASE groups more than that in the model group. The number of apoptotic cells in the model group was significantly higher than that in the normal group by the analysis of apoptotic cell counts (*p* < 0.05), indicating that H_2_O_2_ caused apoptosis in RAW264.7. Compared with the model group, the NASE drug-treated group was able to decrease the apoptosis rate, especially in the high-dose group, and the difference was significant in the apoptosis rate compared with the model group (*p* < 0.05) (Figure 2(b)). No dose-dependent effects of NASE were observed in the Giemsa staining experiments, but different concentrations of NASE were found to have good effects.

### 3.3. Determination of Intracellular ROS Level

**Figure 3:**
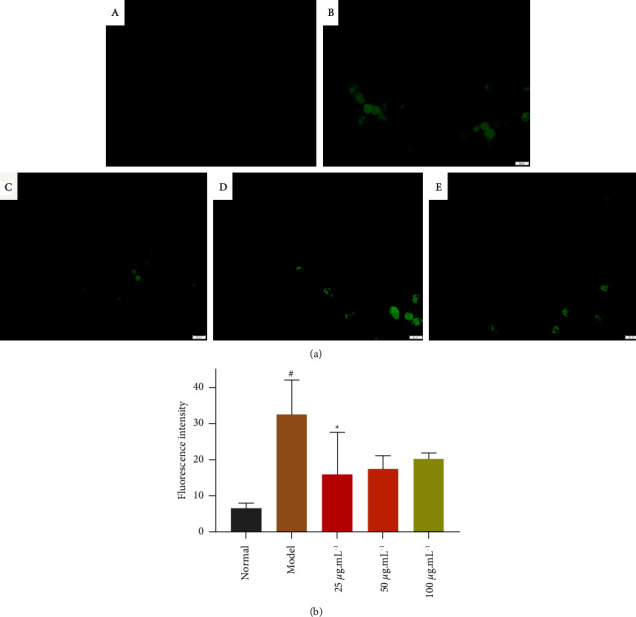
(a) RAW264.7 intracellular reactive oxygen species detection. (b) Graph of fluorescence intensity. (A) Normal group. (B) Model group. (C) NASE low dose group (25 *μ*g·mL^−1^). (D) NASE medium dose group (50 *μ*g·mL^−1^). (E) NASE high dose group (100 *μ*g·mL^−1^). Note: Values are mean ± standard deviation. ^*∗*^Significant difference at the 5% level compared with the model groups. ^#^Significant difference at the 5% level in comparison with the normal group.

ROS is inseparable from the generation of cellular oxidative stress. After H_2_O_2_ stimulated RAW264.7 cells for 2 h, the DCFH-DA probe was added to 96-well plates and incubated for 30 minutes. Then, the probe could enter the cells and react with ROS, and the green fluorescence could be observed under the blue channel of the fluorescence microscope. As shown in Figure 3(a), when RAW264.7 cells were induced with H_2_O_2_ for 2 h, the green fluorescence was significantly increased in the model group, and the green fluorescence in the NASE-treated groups showed a decreasing trend compared with the model group. The results of the quantitative analysis showed that the NASE-treated group reduced the production of ROS compared with the model group, and the low-dose group reached a significant difference compared with the model group (*p* < 0.05) (Figure 3(b)). The difference in fluorescence density between the middle-dose and high-dose groups was not significant compared with the model group (*p* > 0.05). During the experiment of taking photos, we found there is a difference in the density of cells in each field of view, which may lead to a difference in the density of fluorescence. Although we have taken the method of averaging multiple fields of view, the results may still be affected. It may be due to the error of the experimental method, the effects of the medium and high-dose groups were lower than those of the low-dose group compared with the model group. When the ROS content of the environment of the cells is too high, it will lead to an imbalance in the original oxidative state of the cells, resulting in apoptosis. NASE-treated RAW264.7 cells can significantly improve the ability of the cell to scavenge ROS, thus improving the antioxidant capacity of the cells and protecting them. Since no dose-dependent effects of NASE were observed in the Giemsa staining experiments as well.

### 3.4. Flow Cytometry to Detect the Effect of NASE on Apoptosis

**Figure 4:**
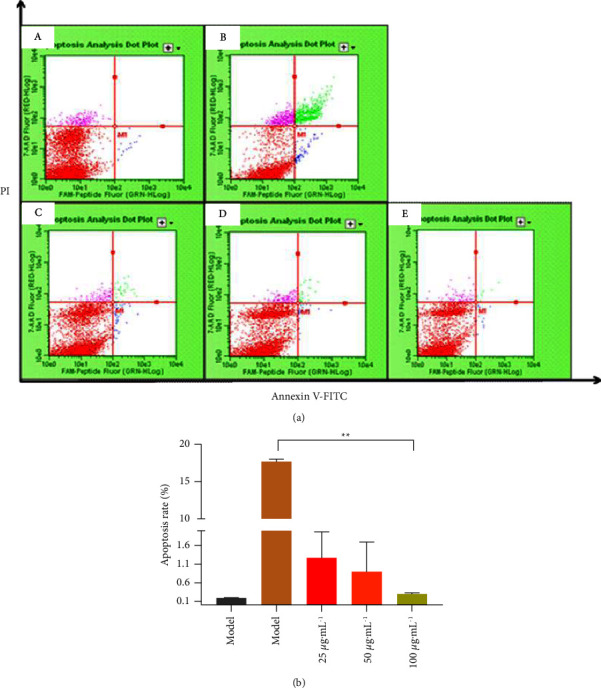
Effects of different concentrations of NASE on H_2_O_2_-induced RAW264.7 cell apoptosis by flow cytometry: (a) RAW264.7 cell apoptosis by flow cytometry. (b) Quantitative analysis of the apoptosis rate. (A) Normal group. (B) Model group. (C) NASE low dose group (25 *μ*g·mL^−1^). (D) NASE medium dose group (50 *μ*g·mL^−1^). (E) NASE high dose group (100 *μ*g·mL^−1^). Note: Values are mean ± standard deviation. ^*∗∗*^Extremely significant difference at the 1% level.

To investigate how NASE inhibits the apoptosis of RAW264.7 cells, a flow cytometry assay was used to examine the cellular apoptosis status. In the experiment, H_2_O_2_ stimulated RAW264.7 cells for 2 h to induce oxidative stress and apoptosis, to evaluate the apoptosis of NASE-treated cells under oxidative stress. As shown in Figure 4, compared with the normal group (apoptosis rate of 0.2% ± 0.84%), the apoptosis rate of the model group (17.02% ± 0.41%) was significantly increased (*p* < 0.01). After a period of NASE pretreatment, the apoptosis rate of cells decreased, respectively. There was a significant decrease in the apoptosis rate among cells treated with high doses of NASE with the model group (*p* < 0.01).

### 3.5. The Body Weight and Liver Index

**Figure 5:**
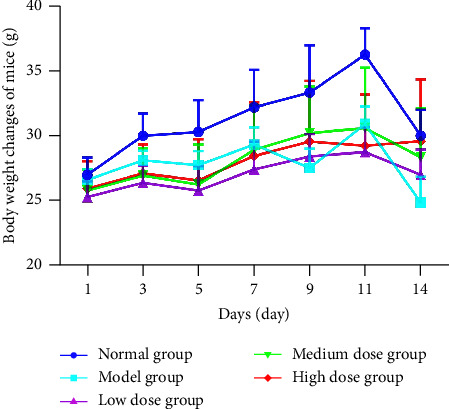
Change of body weight of mice.

Carbon tetrachloride (CCl_4_) can induce chemical liver injury, which is used to test the therapeutic effect of drugs in experimental animals. Intraperitoneal injection of CCl4 was able to cause liver inflammation, resulting in reduced food intake and weight loss in mice. The mice showed liver enlargement and increased liver mass, which in turn manifested as a larger liver index. The changes in the body weight of mice are shown in Figure 5. On days 1–11, the body weight of mice increased gradually in all groups. The body weight of the normal group had the greatest weight gain compared to the other groups. The greatest fluctuations in weight gain were observed in the model group, which may be related to CCl_4_ administration. The weight gain in the NASE-treated groups was lower than that in the normal group. The weight loss on day 14 was related to the 12 h of fasting before the end of the experiment. There was no statistically significant difference in body weight between the model and NASE-treated groups (*p* > 0.05). This finding indicated that the NASE within the measured dosage range had no obvious effect on the body weight of mice. Changes in the liver indexes of mice are listed in Table 2, the liver index of the model group was significantly higher than that of the normal group (*p* < 0.01), indicating that CCl_4_ caused liver swelling in model mice. The liver index of the NASE-treated group was significantly lower than that of the model group (*p* < 0.01, *p* < 0.05), indicating that NASE decreased liver swelling. This result revealed that the liver injury induced by CCl_4_ in mice was successfully modeled.

### 3.6. HE Staining

**Figure 6:**
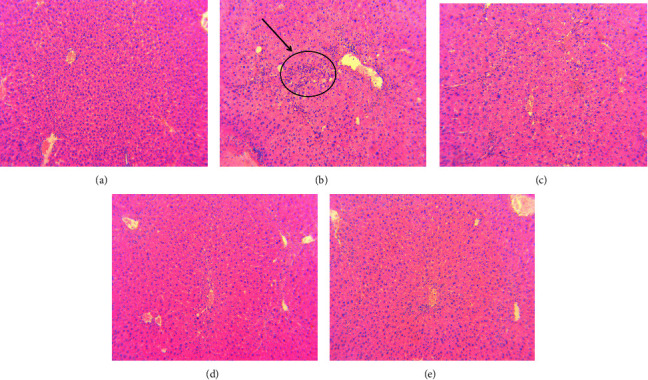
HE staining in the liver (20×). (a) Normal group. (b) Model group. (c) NASE low dose group (20 mg·mL^−1^). (d) NASE medium dose group (40 mg·mL^−1^). (e) NASE high dose group (80 mg·mL^−1^). Note: Values are mean ± standard deviation.

The basic principle of hematoxylin eosin staining (HE staining) is that hematoxylin is a basic stain that stains the nucleus blue and eosin is an acidic stain that stains the cytoplasm purplish red, thereby confirming the basic cell structure. In this study, HE staining was used to analyze the pathological sections of the liver of mice with liver injury. The HE staining was observed and analyzed under a light microscope and it was shown that the liver cells of mice in the normal group were structurally complete, neatly arranged, and had no inflammatory infiltration. The liver cells of mice in the model group were arranged in disorder, the cells were enlarged or deformed, and there were inflammatory infiltration and necrosis. The hepatic cells of the mice in the NASE groups were relatively intact and arranged more neatly than those in the model group. However, there was still some infiltration of inflammatory cells. The overall condition and morphology of the cells improved with the increase in the NASE dosage, as shown in Figure 6.

### 3.7. TUNEL Staining Assay

**Figure 7:**
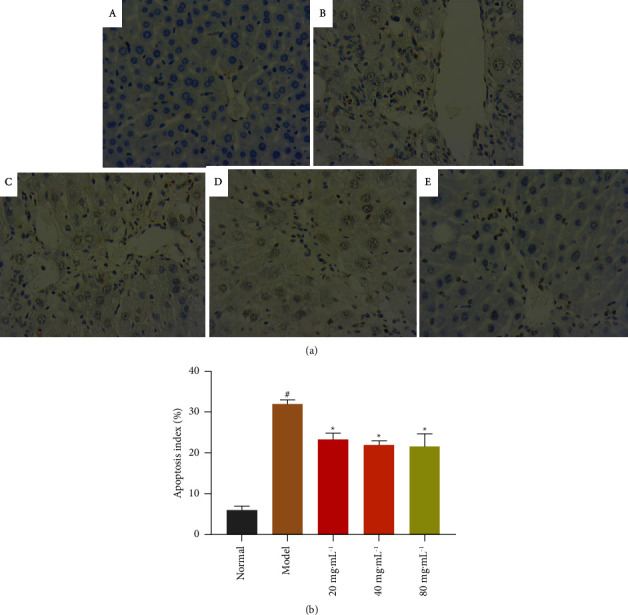
(a) TUNEL method in situ detection of cell apoptosis in liver tissue sections (40×). (b) Graph of the apoptosis rate. (A) Normal group. (B) Model group. (C) NASE low dose group (20 mg·mL^−1^). (D) NASE medium dose group (40 mg·mL^−1^). (E) NASE high-dose group (80 mg·mL^−1^). Note: values are mean ± standard deviation. Compared with the model group, ^*∗*^significant difference at a 5% level. Compared with the normal group, ^#^significant difference at the 5% level.

During apoptosis, chromosomal DNA breaks to form apoptotic vesicles, and TUNEL staining is a combined molecular biology and morphology research method that can stain in situ in intact individual apoptotic nuclei or apoptotic vesicles, which can accurately reflect the typical biochemical and morphological characteristics of apoptosis. Therefore, TUNEL staining was performed on the liver tissues of mice in each group (Figure 7(a)) to observe the formation of apoptotic bodies (tan granules) in liver cells. The results showed that almost no apoptotic bodies were observed in the normal group. Compared with the normal group, the apoptotic bodies in the model group were significantly increased, while the apoptotic bodies in the liver cells of mice in different doses of NASE groups were significantly reduced (Figure 7(b)). This indicated that the apoptosis of liver cells induced by CCl_4_ could be alleviated to a certain extent under the action of NASE.

### 3.8. Expression of Bcl-2, Bax Proteins in Liver Tissue

**Figure 8:**
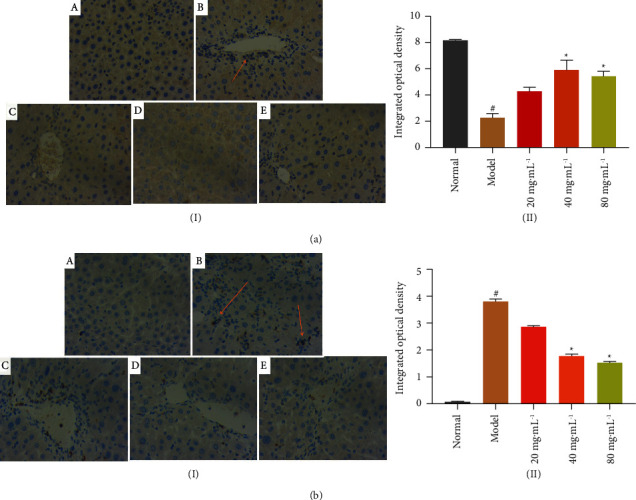
Expression of Bcl-2 and bax protein in liver tissue (40×). ((a)-(I)) Bcl-2 protein expression. ((a)-(II)) Graph of the integrated optical density of Bcl-2 level. ((b)-(I)) Bax protein expression. ((b)-(II)) Graph of the integrated optical density of bax level. (A): Normal group. (B): Model group. (C): NASE low dose group (20 mg·mL^−1^). (D): NASE medium dose group (40 mg·mL^−1^). (E): NASE high dose group (80 mg·mL^−1^). Note: values are mean ± standard deviation. Compared with the model group, ^*∗*^significant difference at a 5% level. Compared with the normal group, ^#^significant difference at the 5% level.

Liver injury causing hepatic fibrosis is a pathological process in which persistent liver injury leads to increased synthesis of extracellular matrix and an imbalance in its degradation, resulting in the development of hepatic fibrosis. Throughout this process, the focus is on the activation of hepatic stellate cells (HSC). There is evidence that there is a class of apoptotic mediators in HSC: Bcl-2 and Bax, with Bax being the main regulator of Bcl-2 activity. Overexpression of Bax in hepatocytes can lead to disruption of mitochondrial integrity. If this action is prevented at the early stage of liver injury, it can play a role in protecting hepatocytes against liver injury. Therefore, in this study, the apoptosis of hepatocytes was regulated by modulating the Bcl-2/Bax pathway, as shown in Figure 8. By measuring the relative levels of the pair of apoptotic genes, Bcl-2 and Bax, it was found that different doses of NASE could reduce the expression of Bax during apoptosis, which could increase the expression of the antiapoptotic gene Bcl-2 and showed a dose-dependent response, indicating that NASE could inhibit the apoptotic effect of CCl_4_ on hepatocytes. It is suggested that NASE can regulate the expression of pro-apoptotic factor Bax and antiapoptotic factor Bcl-2 in the liver as a way to reduce the necrosis of hepatocytes. The results obtained in this study are similar to those of Wang et al. [17], where Ginkgo biloba extract inhibited hepatocyte apoptosis by downregulating Bax, upregulating Bcl-2, and subsequently inhibiting the activation of Caspase3, thereby reducing liver fibrosis. Hu et al. in their study also demonstrated that specnuezhenide inhibited oxidative stress by activating Nrf2 signaling and reducing hepatocyte apoptosis, thereby reducing CCl_4_-induced liver injury in mice [18].

### 3.9. The Content of ALT, AST, ALP, And LDH in Serum

**Figure 9:**
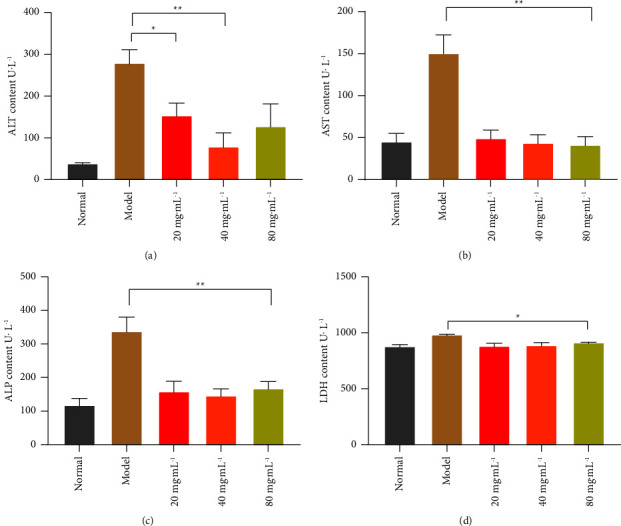
The content of ALT, AST, ALP, and LDH in serum. (a) ALT content. (b) AST content. (c) ALP content. (d) LDH content. Note: values are mean ± standard deviation. ^*∗*^Significant difference at the 5% level. ^*∗∗*^Extremely significant difference at the 1% level.

When hepatocytes are injured, their cell membrane permeability increases and the cytoplasm releases alanine aminotransferase (ALT) and aspartate aminotransferase (AST), and the activity of both in serum will be significantly increased, which is an important index to evaluate the degree of liver injury [19]. The production of cytotoxicity is another manifestation of hepatocyte injury, and lactate dehydrogenase (LDH) is a widely expressed enzyme whose release has been used as a marker of membrane damage. Alkaline phosphatase (ALP) is also widely used as a biochemical indicator of hepatocyte damage [20]. The activity levels of ALT, AST, ALP, and LDH reflect the degree of damage to liver cells. The effects of NASE on serum ALT and AST activities of liver-injured mice are shown in Figures 9(a) and 9(b). Compared with the normal group, the serum ALT and AST activities of mice in the model group were significantly higher (*p* < 0.01), indicating that a model of CCl_4_-induced liver tissue injury had been established. The effects of NASE on the serum ALP and LDH activities of mice with liver injury are shown in Figures 9(c) and 9(d). The serum ALP and LDH activities of mice in the model group showed an increase compared with the normal group. Compared with the model group, the serum ALP and LDH activities of mice in different dose groups were significantly reduced (*p* < 0.01 or *p* < 0.05). The results indicated that NASE treatment can alleviate hepatocyte damage caused by oxidative stress.

### 3.10. The Contents of Intracellular SOD and MDA

**Figure 10:**
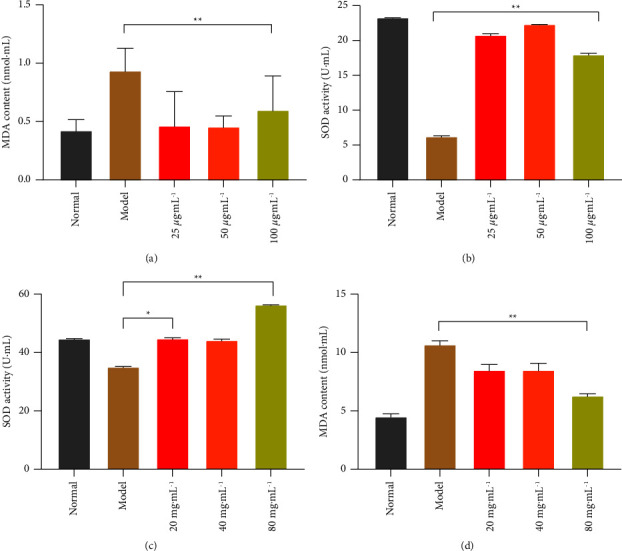
SOD and MDA activity assay. (a) MDA activity in RAW264.7. (b) SOD activity in RAW264.7. (c) SOD activity in liver tissue. (d) MDA activity in liver tissue. Note: values are mean ± standard deviation. ^*∗*^Significant difference at the 5% level. ^*∗∗*^Extremely significant difference at the 1% level.

Superoxide dismutase (SOD) and malondialdehyde (MDA), as important regulators of oxidative stress, account for a large proportion of the evaluation of the body's antioxidants. SOD is the main enzyme and antioxidant for scavenging oxygen radicals in the body, and its activity and content can indirectly reflect the ability of the body to scavenge free radicals and whether the oxidative status of the body is balanced. The content of MDA can reflect the degree of tissue lipid peroxidation in the body, thus indirectly reflecting the degree of oxidative damage in tissue cells. As shown in Figures 10(a) and 10(b), after the cells were induced by H_2_O_2_, the MDA content in the model group increased significantly compared with the normal group, and all dose groups of NASE reduced the production of MDA under oxidative stress, and the difference was highly significant compared with the model group (*p* < 0.01). Meanwhile, the antioxidant enzyme activity of SOD in RAW264.7 cells in the model group was reduced compared with the normal group, and the difference was highly significant compared with the NASE dose groups (*p* < 0.01). As shown in Figures 10(c) and 10(d), the content of SOD activity in the liver tissues of mice in the model group was significantly reduced and the content of MDA increased compared with the normal group (*p* < 0.01). Compared with the model group, the SOD activity content in the liver tissue of mice in each dose group of NASE increased significantly and the MDA content decreased, with the most significant increase in the SOD activity content (56.4 U/mL^−1^) and the most significant decrease in the MDA content (6.3 nmol·mL^−1^) of mice in the high dose group of NASE (*p* < 0.01).

### 3.11. Quantitative Real-Time Polymerase Chain Reaction

**Figure 11:**
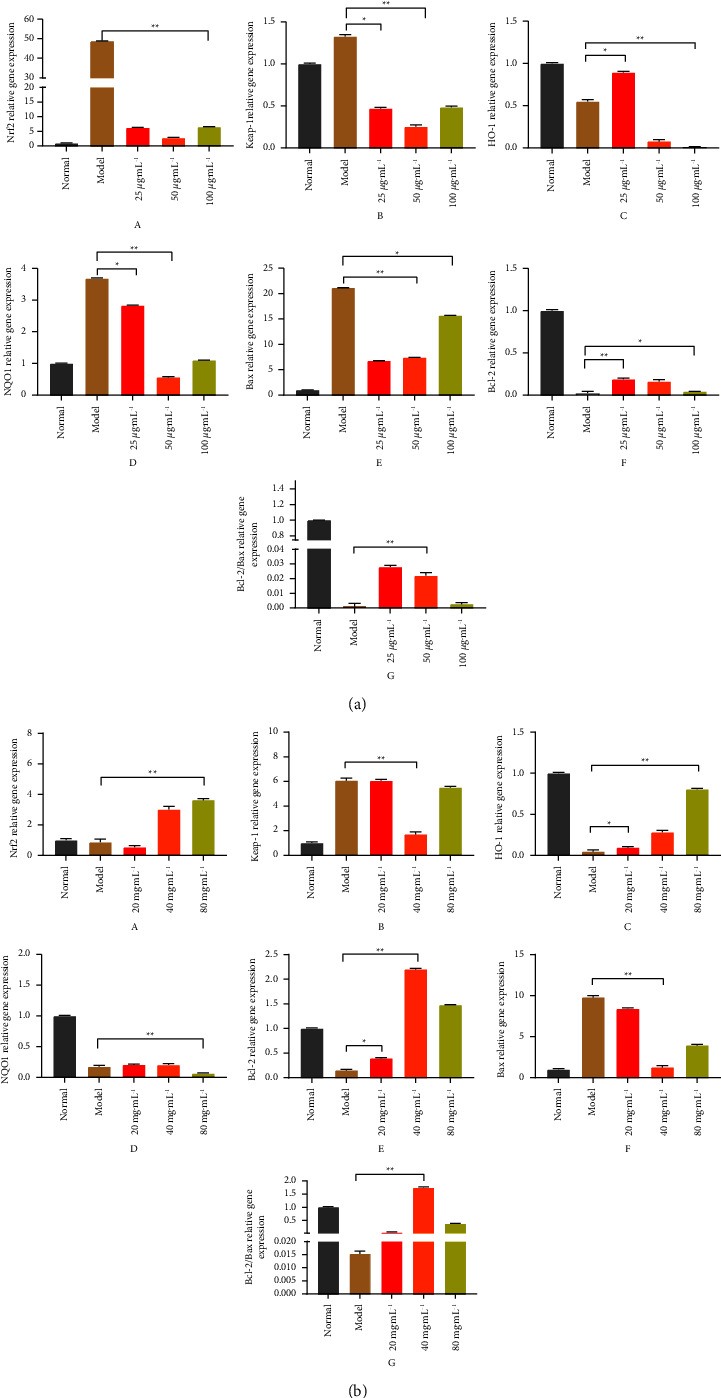
Real-time PCR assay. (a) RAW264.7 cells. (b) Liver issue. (A) Nrf-2 relative gene expression. (B) Keap-1 relative gene expression. (C) HO-1 relative gene expression. (D) NQO-1 relative gene expression. (E) Bcl-2 relative gene expression. (F) Bax relative gene expression. (G) Bcl-2/Bax relative gene expression. Note: Values are mean ± standard deviation. ^*∗*^Significant difference at the 5% level. ^*∗∗*^Extremely significant difference at the 1% level.

The Nrf2/HO-1 pathway is an antioxidant signaling pathway that the body protects normal cells against oxidative stress, as well as exogenous damage. When hepatocytes are stimulated by CCl_4_, Nrf2 phosphorylates into the nucleus and binds to the DNA sequence of ARE, an antioxidant element in the nucleus, initiating the expression of ARE-regulated downstream phase II detoxification enzymes and antioxidant enzyme genes, thereby resisting oxidative stress-induced damage. Therefore, in this experiment, we analyzed the transcript levels of Nrf-2, HO-1, Keap-1, NQO-1, Bax, and Bcl-2 mRNA in RAW264.7 cells and liver tissues by RT-PCR to investigate the effect of NASE on the Nrf2/HO-1 pathway, and the results are shown in Figure 11. Accordingly, it was observed that Nrf-2, Keap-1, and NQO-1 expressions were downregulated, and HO-1 expressions were upregulated in RAW264.7 cells of NASE groups compared to the model group (*p* < 0.05). Keap-1 and NQO-1 expressions were downregulated, and Nrf-2 and HO-1 expressions were upregulated in the liver tissue of NASE groups compared to the model group (*p* < 0.05). Interestingly, the expression levels of Nrf-2 show the opposite trend in RAW264.7 and liver tissue. Bcl-2 and Bax's genes are apoptosis-related genes discovered in recent years, and they are closely related to the occurrence, development, and prognosis of cells. The expression results of Bcl-2 and Bax's genes in each group are shown in Figure 10. Compared with the normal group, the Bax gene expression level in the model group was significantly upregulated, and the Bcl-2 gene expression level in the model group was significantly downregulated in RAW264.7 cells and mice liver tissue (*p* < 0.05); compared with the model group, the Bax mRNA expression levels in the NASE groups were significantly downregulated, and the Bcl-2 gene expression level in the model group was significantly upregulated in RAW264.7 cells and mice liver tissue (*p* < 0.05). The ratio between the Bcl-2/Bax proteins is a key factor in regulating cell apoptosis, and the increase in the Bcl-2/Bax ratio can inhibit the occurrence of apoptosis. After NASE treatment, the Bcl-2/Bax ratios all were improved in RAW264.7 cell and liver tissue. The inhibitory effect of NASE on the cell oxidative stress and apoptosis may be associated with the activation of the Nrf2/Keap1//HO-1 and Bax/Bcl-2 signaling pathways.

## 4. Discussion

When oxidative stress occurs in the body, the balance of cellular oxygen utilization is disrupted, resulting in the accumulation of ROS in vivo [21]. Excessive intracellular ROS will cause cellular damage, loss of function, and ultimately inflammation and even apoptosis. The liver is an important metabolic organ in the body and plays a role in various pathways such as macronutrient metabolism, immune response, and the decomposition of biological compounds [22]. CCl_4_ is commonly used to establish an experimental model of acute liver injury [23] and CCl_4_ is metabolized by the liver to generate the free radical trichloromethyl [24]. The free radicals generated after CCl_4_ are metabolized by the liver, as a result of the free radical chain reaction, lipid peroxidation of the cell membrane is directly induced, and the cell membrane is damaged, resulting in oxidative stress, degeneration, liver cell damage, and necrosis.

Numerous experiments have demonstrated that AS has strong antioxidant and anti-inflammatory activities in vivo [25–28]. The NASE is the active ingredient from the rhizome of the *A*. *senticosus*. In this experiment, NASE can promote the proliferation of RAW264.7 cells, reduce intracellular ROS level and MDA content, and increase SOD activity. In CCl_4_-induced liver injury, the contents of ALT, AST, ALP, and LDH are an evaluation indicator of liver function. The NASE can increase their levels in serum, which are agreeing with previous reports [29–32]. The histopathological findings confirm the experimental results. After NASE treatment, a large number of inflammatory cells infiltrated the lobular structure, and bleeding was significantly reduced. There has been previous research reporting that AS can alleviate liver injuries in many animal models, and the present results confirm that [33–35].

In the cells, Nrf-2 regulates the expression of genes relating to cell defense against oxidative stress [36]. In physiological conditions, Nrf-2 binds to Keap1 to remain in the cytoplasm until it is degraded by the proteasome to maintain its low levels of protein [37]. The Nrf-2 protein is released from Keap1 upon oxidative stress and translocated into the nucleus. By binding to antioxidative response elements (AREs), Nrf-2 hetero-dimers induce the transcription of related antioxidant enzymes, such as SOD, HO-1, and NQO-1 [38]. Apoptosis of cells is regulated by Bcl-2 and Bax, two signaling pathways within mitochondria [39]. Normally, Bcl-2 inhibits apoptosis, while Bax promotes apoptosis. When Bcl-2 is deficient, apoptosis may occur. In this work, the NASE can upregulate the gene expression of Bcl-2 and HO-1 (*p* < 0.05), and downregulate the gene expression of Bax, Keap-1, and NQO-1 (*p* < 0.05). Thus, NASE can inhibit the oxidative stress of cells through the Nrf2-related pathway Keap1-Nrf2-ARE, and reduce the apoptotic activity of cells through the Bax/Bcl-2 signal pathway.

## 5. Conclusions

In a word, current results verified that NASE protected RAW264.7 cells from H_2_O_2_-induced oxidative stress and prominently ameliorated CCl_4_-induced liver damage. Therefore, NASE has the potential to be a therapeutic medicine for preventing and treating cell damage and liver damage caused by oxidative stress.

## Figures and Tables

**Table 1:** Sequence of primers of QT-PCR.

Gene	Primer sequences (5′-3′)
Nrf2	F: ACATGGAGCAAGTTTGGCAG
	R: TGGAGAGGATGCTGCTGAAA

Keap-1	F: AGCGTGGAGAGAGATATGAGCC
	R: ATCATCCGCCACTCATTCCT

HO-1	F: TGAGAGGAACCAAGTGTTTGAG
	R: CAGGGGGACTTTAGCTTTAGAA

NQO1	F: ATTGTATTGGCCCACGCAGA
	R: GCACTCTCTCAAACCAGCCT

Bax	F: ATTTCCAGACACCGAGGG
	R: TAAGCCAAATGTAGCAAGG

Bcl-2	F: CGGGAGAACAGGGTATGA
	R: CAGGCTGGAAGGAGAAGAT

GAPDH	F: GAAGGTCGGAGTCAACGGAT
	R: CCTGGAAGATGGTGATGGG

**Table 2:** Changes in the liver index in mice.

Group	Dose (mg·mL^−1^)	Number	Liver index (%)
Normal group	—	8	4.1 ± 0.42
Model group	—	8	6.2 ± 0.5^##^
NASE low dose	20	8	4.6 ± 0.27^*∗∗*^
NASE medium dose	40	8	4.5 ± 0.57^*∗∗*^
NASE high-dose	80	8	5.4 ± 1.2^*∗*^

Values are mean ± standard deviation. Compared with the model group, ^*∗*^significant difference at the 5% level. ^*∗∗*^Extremely significant difference at the 1% level. ^##^indicates a significant difference at the 1% level.

## Data Availability

The data sets used and/or analyzed during the present study are available from the corresponding author on reasonable request.
